# Considerations and Practical Options for Measuring Muscle Strength: A Narrative Review

**DOI:** 10.1155/2019/8194537

**Published:** 2019-01-17

**Authors:** Richard W. Bohannon

**Affiliations:** Department of Physical Therapy, College of Pharmacy and Health Sciences, Campbell University, Lillington, NC, USA

## Abstract

Muscle strength impairments are related to mobility limitations and other untoward outcomes. This narrative review, therefore, describes considerations relative to the definition and measurement of muscle strength. Thereafter, practical options for measuring muscle strength are described and their clinimetric properties are delineated. Information provided herein may help students, clinicians, and researchers select the strength tests best suited to their research needs and limitations.

## 1. Introduction

Muscle strength, a “muscle power function” according to the International Classification of Functioning, Disability, and Health [1] is defined herein as the maximum voluntary resultant output that muscles can bring to bear on the environment under a specific set of test conditions [2]. Muscle strength is an important body function that decreases with age in adults [3] and is impaired in diverse medical conditions including stroke [4], spinal cord injury [5], motor neuron disease [6], multiple sclerosis [7], myopathy [8], Parkinson's disease [9], chronic obstructive pulmonary disease [10], heart failure [11], peripheral arterial disease [12] arthritis [13] infection, [14] and alcoholism [15]. It is also impaired after major surgery [16]. Impairments in muscle strength are noteworthy as they can contribute to mobility limitations [17–20] and serve as a predictor of important outcomes such as mortality [21] hospital length of stay [22], and hospital readmission [23]. In light of these facts, practical options for measuring muscle strength are needed. The purpose of this paper is to review some important considerations relative to the definition of muscle strength and its measurement and to discuss practical options for measuring muscle strength.

Several considerations relative to the definition of muscle strength used in this review require elaboration. First, with strength, the muscular output must be maximum and voluntary. Maximum does not necessarily imply that the measured output is the most that can be achieved during a single effort. It simply means that it is the most that is voluntarily achieved when the test is conducted as intended. For example, either a one-repetition maximum load or a 7-10 repetition maximum load could be used to indicate the strength of the knee extensors [24], so long as the knee extension is voluntary. Involuntary output of the knee extensors resulting from an external stimulus such as electrical stimulation [25] would not be considered to be strength in this review. Second, the output being measured is usually the result (summed effect) of the activation of numerous muscles—some quite distant. For example, shoulder abduction involves output from ipsilateral shoulder muscles (deltoids and supraspinatus) and scapular rotators (trapezius and serratus anterior), but output from the contralateral lateral trunk flexors contributes as well [26]. Consequently, it is typically the strength of actions (e.g., shoulder abduction) rather than individual muscles or muscle groups (e.g., middle deltoid) that is actually being measured and will be emphasized hereafter. Third, the muscle output must be brought to bear on the environment. The elbow flexors are clearly acting on the environment when an individual performs a “biceps curl” with a dumbbell. The same individual, however, is also acting on the environment when using the diaphragm to inhale air or the pelvic floor muscles to maintain urinary continence. Muscles crossing an ankylosed joint, on the other hand, are not acting on the environment. The force they generate makes no difference. Finally, the maximum output produced will differ with test conditions. For example, the maximum force generated by the hamstrings is much greater when the hip is flexed (and the hamstrings are elongated) than when the hip is extended (and the hamstrings are shorter) [27].

Regardless of the practical option used to measure muscle strength, there are considerations that must be addressed. First, the effects of gravity must be considered. If an action such as knee extension is measured against gravity, as it could be during seated testing, the actual output of the knee extensors would be the torque they generate to move the mass of the leg against gravity plus whatever additional torque they can generate against external resistance [28]. The heavier and longer the segment being moved, the greater the output required to move or hold it against the pull of gravity [29]. Grading schemes used in manual muscle testing (MMT) often take the effect of gravity into account; measurements of strength obtained by other means should as well [30]. Second, adequate stabilization must be provided. In the absence of such stabilization the full output of the muscles of interest (e.g., knee extensors) may not be captured [31] or extraneous movements may contribute inadvertently to the resultant output measured (e.g., scapular elevation during elbow flexion). Third, the point of resistance can affect strength measures. For example, if the hip abduction force measured 0.5 meters from the hip is 20kg, the force measured 1.0 meter from the hip will be 10 kg. Ideally, therefore, the point of output measurement should be consistent and in line with procedures used to establish norms. Fourth, the same types of testing contractions (i.e., eccentric, concentric, isometric (make and break)) should be used when comparing strength measurements between sessions or with reference values. This is because maximal eccentric (lengthening) outputs tend to exceed maximum concentric (shortening) outputs [25] and maximum isometric outputs are greater with break tests than with make tests [32].

## 2. Practical Options for Measuring Muscle Strength

In the context of this review a practical option is one that is inexpensive, portable, quick, and easily performed. The specific options considered practical in this review, therefore, are manual muscle testing, field tests, hand-held dynamometry, and hand-grip dynamometry. Isokinetic and fixed dynamometry, weight determined strength (e.g., 1 repetition maximum), and patients' assessments of their own strength are not considered in this review. A description of each practical option as well as a brief discussion of its clinimetrics will be presented. The clinimetrics to be addressed herein are reliability, validity, responsiveness, and interpretability.

### 2.1. Manual Muscle Testing

MMT has been in use for more than a century [33]. It involves the use of observation, palpation, and force application by an examiner to determine the strength of a muscle action. In the absence of movement, palpation and observation are used to discern whether muscles of interest are active. In the presence of movement, observation is used to estimate the proportion of an action's test range that is completed. Where movement through the full test range is possible, examiner applied break-test force is used to grade the magnitude of muscle output.

There are several well accepted approaches to MMT; chief among the approaches are those of the Medical Research Council [34], Kendall et al. [35], and Daniels and Worthingham [36]. The grading schemes associated with these approaches are consistent in that all 3 assign a minimum score of 0 when there is no contraction or activity noted and a maximum score of 5 when strength is “normal.” However, the approach of Kendall et al, unlike the approaches of the Medical Research Council and Daniels and Worthingham uses plus and minus designations to more precisely grade muscle strength. Kendall et al and Daniels and Worthingham suggest qualitative scores that can be used in lieu of numerical scores. Specifically they indicate that the scores of trace, poor, fair, good, and normal can be used instead of 1, 2, 3, 4, and 5 (respectively). This I strongly discourage as qualitative scores can grossly misrepresent the magnitude of muscles' output. Beasley long ago showed that knee extension strength was often graded “normal” when dynamometry showed it to be only about 50% of normal [37]. Clearly, referring to such strength as normal is a misappropriation of the word. More recently, Dvir concluded that elbow and knee actions graded as 4 (good) may be generating as little as 10% of expected maximum output [38]. Calling such output “good” grossly exaggerates the strength being described.

Table 1 presents the standard numerical scheme recommended herein for grading most muscle actions. It has been presented previously in the rehabilitation literature [39, 40]. The scheme involves no qualitative distinctions and makes no reference to normal. I would argue that in the absence of normative values for manual testing, grades should be based on the magnitude of the break force withstood by the tested individual. So, a 20-year-old male gymnast may demonstrate shoulder abduction strength of 5/5 because he can hold against a maximum breaking force without giving way, whereas a 76-year-old sedentary woman may demonstrate shoulder abduction strength of 3+/5 because she held against only a minimum breaking force before giving way. An experienced tester should not be surprised by these grades- they support the ability of MMT to differentiate between the strengths of a younger athletic man and an older woman. If the tester were to give the older woman the maximum score of 5/5 because her performance seemed “normal” for age and gender, the ability to distinguish between her and the gymnast would be lost. An addition to the standard grading scheme presented in Table 1 is what has been referred to as the “Most Common Alternative Scheme.” This scheme (Table 2) is applicable to muscle actions where gravity has a minimal effect on strength measurements (e.g., finger actions) or where actions are tested without altering the effects of gravity (e.g., neck flexion).

The strength of numerous specific muscle actions (e.g., shoulder abduction) can be graded using MMT. Although the addition of multiple ordinal MMT scores is inappropriate from a statistical standpoint, composite scores of multiple actions (e.g., hip flexion, knee extension, and ankle dorsiflexion) are often derived with the intent of characterizing muscle strength. Composite scores of 3 to 10 muscle actions developed for specific diagnostic groups (Table 3) include the Motricity Index (stroke) [41], Motor Index Score (spinal cord injury) [42], MRC Sum Score (neuropathy) [43], and MMT 8 (myositis) [44].

The clinimetric properties of MMT have been studied extensively. Numerous studies have described the test-retest and inter-tester reliability for a large number of specific muscle actions. In a review of these studies, Bohannon found that pairwise reliability (weighted kappa) coefficients were usually “substantial” or “almost perfect” but could also be extremely low [45]. He recommended, therefore, that reliability should be confirmed rather than assumed before using MMT in clinical or research settings. The subjectivity of tester resistance [46] and differences in tester strength [47] are likely contributors to limitations in reliability- particularly among higher scores. Problems with the use of composite scores notwithstanding, their reliability tends to be better than that of scores of individual muscle actions [8].

The validity of MMT is supported by reports of significant correlations between MMT scores and measurements obtained by dynamometry. For example, Bohannon reported large curvilinear relationships between knee extension strength measured by MMT and by hand held dynamometry in an acute rehabilitation setting (R=0.887) [40] and between grip strength measured by MMT and by hand-grip dynamometry in a home-care setting (R= 0.840 and 0.934) [48]. The validity of MMT is also supported by significant correlations between MMT scores and performance at functional activities such as sit-to-stand and gait [18, 49]. These correlations notwithstanding, MMT has limited sensitivity as a measure of muscle strength, particularly among individuals whose strength is not particularly impaired [50, 51].

Information on the responsiveness and interpretability of manual muscle testing scores is woefully absent. Typical responsiveness statistics (e.g., minimal detectable change) do not apply to MMT scores obtained for specific muscle actions as the scores are ordinal in nature. Nevertheless, studies focused on patients with spinal cord injuries have clearly shown that MMT is capable of identifying strength increases over the course of weeks or months postinjury. Mange et al., for example, showed that in the “zone of partial preservation” 100% of patients recovered 1 MMT grade within a median time of 0.5 or 1 month postinjury and 86% or more of patients recovered 2 MMT grades within a medium time of 3 months [52]. Clearly, this responsiveness does not apply to patients with scores of 5/5 for whom large increases or decreases in strength are not accompanied by altered MMT scores. There is no information available to assist in interpreting MMT scores. Both Kendall et al. [35] and Daniels and Worthingham [36] have recommended that potential examiners test numerous individuals to get a sense of normal, but neither suggest normal scores for specific actions of normal individuals.

### 2.2. Field Testing

Herein field tests refer to tests that use body weight as resistance and time or repetitions as the primary means of quantifying performance. Although many such tests have been described in the literature, only the sit-to-stand (STS) and heel-raise (HR) tests will be addressed hereafter.

#### 2.2.1. Sit-to-Stand Tests

The STS test is meant to characterize the strength of the lower limbs. The test, which typically incorporates a standard height armless chair, involves either documenting the time required to complete a given number of repetitions (usually 5) or counting the number of repetitions completed in a period of time (usually 30 seconds). In either case, patients should begin sitting forward on the chair with their feet flat on the floor. They are to stand up completely and sit down as firmly as fast as possible while their upper limbs are folded across their chests. Repetitions should be counted aloud by the tester.

The 5 repetition STS has been used as a component of the short physical performance battery [53] or as a stand-alone test in hundreds of studies. Timing begins either on the command “go” or when movement first begins and ends either when the fifth stand-up is achieved or the patient sits after the fifth stand-up [54]. The test-retest reliability of the 5 repetition STS test has been estimated across 10 studies to be 0.81 [55]. Although performance on the 5 repetition STS test is correlated with motor control, balance, and sensation, its validity as a measure of lower limb muscle strength is supported by its greater correlation with knee extension strength [56]. Further support for the validity of the 5 repetition STS test is provided by the high correlation of performance on the test and performance on the timed up and go and gait speed tests [57]. Moreover, performance on the test differentiates between individuals with (16.4 seconds) versus without (13.4 seconds) vestibular disorders [58] and between future recurrent and nonrecurrent fallers (15.0 second cut point) [59]. The responsiveness of the 5 repetition STS test has been characterized using the minimal detectable change with values ranging from 2.5 seconds [60] to 3.1 seconds [61] as well as with a minimal clinically important difference of 2.3 seconds for patients with vestibular disorders [62] and 1.7 seconds for patients with chronic obstructive pulmonary disease [63] Bohannon has reported normative values for the test for older adults based on meta-analysis [63]. He suggested that times exceeding 11.4, 12.6, and 14.8 seconds might be considered abnormal for 60 to 69, 70 to 79, and 80 to 89 year-olds, respectively [54].

The 30 second STS test is a component of the Senior Fitness Test. It involves counting the number of STS repetitions performed in 30 seconds. Tested individuals who are more than half-way up at 30 seconds are credited with completing the final repetition [64]. Good to excellent reliability has been reported for the test. Jones et al. reported reliability coefficients of 0.84 and 0.92 for older men and women retested 2 to 5 days after a baseline test [65] Alfonso-Rosa et al. estimated the test-retest reliability coefficient over a 1 week period to be 0.92 for older adults with type 2 diabetes [66] For patients with hip osteoarthritis Wright et al. found an interrater reliability coefficient of 0.81. [67]. Validity of the test is supported by its correlation with “leg-press” strength (0.78 for men and 0.71 for women) [65]. Its validity is further supported by more repetitions being performed by older adults who are younger and more active [65, 67]. The responsiveness of the 30 second STS test is evinced by descriptions of its minimal detectable change- 3 repetitions for patients with Parkinson's disease [68] and 3.3 repetitions for patients with type 2 diabetes [66] and minimal clinically important difference of 2.0 to 2.6 repetitions [67]. Rikli and Jones have provided extensive normative data for interpreting performance of men and women within 5 year age strata on the 30 second STS test [64]. Macfarlane et al. have supplemented that data with information from older Hong Kong Chinese [69].

#### 2.2.2. Heel-Raise Test

The HR test is meant to characterize the strength of the ankle plantarflexor muscles [70]. The test is best conducted while tested individuals stand facing a wall with their hands lightly resting on the wall for balance. Some protocols require that tested individuals stand on a wedge [71]. Tested individuals first do a maximum bilateral HR to help establish test range. They then perform unilateral HRs at a rate of 1 every other second while nonweightbearing on the other lower limb. A metronome can be used to help control the rate. Each complete HR should be counted aloud. Care should be taken to assure that the knee of the tested lower limb remains fully extended. Scoring for the test is as follows: 0 = no evidence of contraction, 1 = evidence of contraction but no movement, 2 = partial range of motion, 3 = full range of motion (1-9 times), 4 = full range of motion (10-19) times, and 5 = full range of motion (20 or more times.) However, the actual number of HRs provides a more specific indication of strength of the ankle plantarflexors.

High test-retest reliability coefficients have been reported for the test when performed by healthy adults (0.93-0.96) [71, 72], patients with heart failure (0.93-0.94) [71], and patients undergoing hemodialysis (0.94-0.97) [73]. The validity of the HR test is supported by significant relationships between performance on the test and gait speed and use of an assistive device during gait [74]. Further evidence for the validity of the test is provided by research showing that the repetitions completed are greater for younger than older adults [75] and by controls than by patients with heart failure [71] or venous insufficiency [76]. The responsiveness of the HR test can be derived from data in several studies. Data presented by Hébert-Losier et al. for healthy adults yield a minimal detectable change (95%) of 6 HRs [72]. Data provided by Segura-Orti et al. demonstrates a minimal detectable change (95%) of 4.4 HRs on the right and 6.1 HRs on the left [73]. Normative values have been posited by several authors. Lunsford and Perry recommended 25 repetitions as a standard for normal after observing a mean 27.9 repetitions by more than 200 male and female adults (20 to 59 years) [70]. Svantesson et al. observed a similar mean number of HRs (n = 25) by 10 healthy women (mean 24 years) [77]. Others have reported higher (mean 32.1 -33.8) [72] or lower (mean 2.7 -22.1) [75] normative values. Jan et al. recommended that performance be interpreted based on age and gender and presented stratified normative values as well as explanatory regression equations [75].

### 2.3. Hand-Held Dynamometry

Hand-held dynamometry (HHD) is a procedure by which a dynamometer held in the hand of a tester is applied to the body of an individual being tested. All testing should be performed gravity lessened/eliminated or corrected (reasoning explained heretofore). The tested individual exerts an increasing (crescendoing) force against the dynamometer over a period of several seconds while the tester holds the dynamometer steady against the effort of the tested individual. Thus, an accommodating isometric make test is performed. Bohannon has thoroughly described HHD testing procedures for numerous muscle actions [78–80]. Adherence to these procedures is strongly encouraged.

Both the test-retest [81] and interrater reliability [82] of measurements obtained by HHD have been studied. Reviews of the studies demonstrate that acceptable reliability is possible, but that it cannot be assumed. As with MMT, the problem centers on inadequate strength of the tester relative to the tested individual. Wikholm et al. demonstrated this clearly by having a weak, moderately strong, and strong tester use HHD to test the strength of a weak (shoulder external rotation), moderately strong (elbow flexion), and strong (knee extension) muscle action [83]. The reliability coefficients for the testers were 0.93 for the shoulder, 0.78 for the elbow, and 0.23 for the knee. Use of a belt to stabilize the dynamometer can substantially improve the reliability of measurements obtained from stronger muscle actions (e.g., knee extension and hip abduction) [84, 85].

The validity of measurements obtained by HHD is dependent, like the reliability of the measurements, on the tester having adequate strength to hold stably against the effort of the tested individual. Without such strength, the maximum force the tester can measure is limited by his or her own strength [86]. The aforementioned notwithstanding, valid measurements of muscle strength can be obtained using HHD. Measurements obtained with the hand-held device have been shown to correlate significantly with those obtained with an isokinetic dynamometers [87, 88] and with the performance of functional activities such as STS [18], gait [89], and stair ascent [90]. Measurements obtained with hand-held dynamometers have also been shown to distinguish between known groups (e.g., healthy adults versus patients with stroke) [91] and known conditions (e.g., fractured versus nonfractured side of patients with hip fracture) [92].

Evidence for the responsiveness of measurements obtained by HHD is notable, at least for knee extension. In a systematic review of 5 studies, Bohannon reported minimal detectable changes that ranged from 7.6 to 92.1 Newtons [81]. In a later study he estimated minimal detectable changes of 46.0 and 57.1 Newtons for patients treated in a home-care setting and 78.6 and 79.0 Newtons for patients treated in acute rehabilitation [93]. Although no formal determination of minimal clinically important difference has been described for hand-held dynamometry, a report by Bohannon provides relevant information [94]. The report focused on adults participating in an inpatient rehabilitation regimen who were initially dependent in rising from a chair. Patients who transitioned to independence in STS over the course of rehabilitation demonstrated a 43% increase in combined knee extension force whereas patients who remained dependent in STS demonstrated a 3% decrease in combined knee extension strength.

Normative reference values are available for interpreting measurements of muscle strength obtained using HHD. Two descriptive studies were the first to provide such values for multiple actions of adults. They used essentially identical procedures but different dynamometers [78, 79]. Others have since published normative values for children and adults [95, 96] and for knee extension [97] and shoulder rotation [98].

### 2.4. Hand-Grip Dynamometry

Hand grip dynamometry, as distinguished from HHD (previously described), is a procedure by which a dynamometer is used to measure a tested individual's grip strength. The procedure is widely employed, not just as an indicator of grip strength itself, but as an indicator of overall strength as well. Though there is some controversy regarding the use of grip strength to characterize overall strength [99, 100], its presumptive value as a sign of generalized weakness has fostered its continued use in the identification of frailty [101], sarcopenia [102], and malnutrition [103]. Grip strength has been described as a vital sign [104] and recommended for routine use in the assessment of older adults admitted to hospital [105].

There are numerous protocols available for measuring grip strength, but that suggested by Roberts et al. is probably the most comprehensive and research informed [106]. They suggested use of a calibrated Jamar dynamometer with its handle in the second handle position with the tested individual sitting with the forearm and wrist in a neutral position and supported on an armrest, the elbow flexed 90 degrees, and the shoulder in 0 degrees abduction and flexion.

The test-retest reliability of grip is well-established. Bohannon systematically reviewed the topic and found that for older adults the reliability coefficient ranged from 0.41 to 1.00, but that in more than 90% of the studies the coefficient was at least 0.80 [107]. Good to excellent test-retest reliability has also been found for patients with stroke [108] and patients undergoing lung transplants [109].

The convergent validity of dynamometer measured grip strength with manual muscle tested grip strength [51] and patient reported upper limb strength [110] has been demonstrated. Grip strength measured with a dynamometer has also been shown to correlate with overall strength and function of the upper limb in patients with stroke [48, 108] and other diseases. Weak grip strength has predictive validity for numerous untoward outcomes, including mortality, postoperative complications, hospital length of stay, discharge disposition, hospital readmission, fractures, and physical functioning [104, 111]. The responsiveness of hand grip strength measured with a dynamometer has been described using the minimal detectable change and minimal clinically important difference. Values for minimal detectable change from 3 different diagnostic groups range from 2.7 to 5.2kg [61, 107, 108]. Minimal clinically important differences identified in a recent systematic review ranged from 0.04 to 6.5kg, but the 6.5 kg value is probably the most legitimate statistically [112].

There is an abundance of normative data for grip strength dynamometry. Some norms are summarized by right and left hand. Other norms are presented by dominant and nondominant hand. In any case, norms are typically presented as summary statistics for specific strata (e.g., gender, side, and age-group) but may also be presented using regression equations. Perhaps the most extensive norms are those derived from more than 100,000 adults by Leong et al. [113]. Those norms are stratified by geographic location as well. Another good source of norms is those derived by meta-analysis by Bohannon et al. [114, 115].

## 3. Discussion

Muscle strength testing is a common component of the physical examination of patients. Herein, muscle strength has been defined and its importance clarified. Factors to be considered in all strength testing have been delineated. Practical strength testing options have been described along with their clinimetric properties. Each option has strengths and limitations. MMT requires no equipment but is influenced by tester judgment and strength; it lacks sensitivity (particularly within the higher grades) and lacks normative values. Field tests are functional and possess good clinimetric properties, but they cannot be completed by patients with extreme weakness. HHD provides objective measures of strength but like MMT can be influenced by tester strength. The cost of hand-held dynamometers may be prohibitive to some. Hand-grip dynamometry has outstanding clinimetric properties but is limited to the measurement of grip strength, which may or may not be adequate as an indicator of overall strength. Consequently, the availability of skilled examiners, instrumentation, and the specifics regarding the pathologies being examined will have to inform decisions regarding the procedures selected for strength testing. In any case, standardized procedures and systematic training will be required before using the procedures described herein.

## Figures and Tables

**Table 1:** Standard grading scheme recommended for scoring most muscle actions.

Grade	Description
0	No muscle action notable by observation or palpation

1	Muscle action observed but no movement noted

1+	With gravity eliminated, movement observed through < 50% of range

2-	With gravity eliminated, movement observed through > 50% of range

2	With gravity eliminated, movement observed through full range

2+	Against gravity, movement observed through <50% of range

3-	Against gravity, movement observed through >50% of range

3	Against gravity, movement observed through full range and test position held

3+	As 3 but able to hold against minimum break force.

4-	As 3 but able to hold against near moderate break force.

4	As 3 but able to hold against moderate break force.

4+	As 3 but able to hold against near maximum break force.

5	As 3 but able to hold against maxumum break force.

**Table 2:** Most common alternative grading scheme applicable to some muscle actions.

Grade	Description
0	No muscle action notable by observation or palpation

1	Muscle action observed but no movement noted

2	Partial range of motion observed

3	Full range of motion observed

3+	As 3 but able to hold against minimum break force.

4-	As 3 but able to hold against near moderate break force.

4	As 3 but able to hold against moderate break force.

4+	As 3 but able to hold against near maximum break force.

5	As 3 but able to hold against maximum break force.

**Table 3:** Muscle actions contributing to 4 composite scores.

Muscle Action	Motricity Index	Motor Index Score	MRC Sum Score	MMT 8
Neck flexors				X

Shoulder abduction	X		X	X

Elbow flexion	X	X	X	X

Elbow extension		X		

Wrist extension		X	X	X

Prehension	X			

Finger flexion (middle)		X		

Finger abduction (5^th^)		X		

Hip flexion	X	X	X	

Hip abduction				X

Hip extension				X

Knee extension	X	X	X	X

Ankle dorsiflexion	X	X	X	X

Great toe extension		X		

Ankle plantarflexion		X		

*∗* Where specific muscles were originally described, corresponding actions have been substituted.
